# ClpL Chaperone as a Possible Component of the Disaggregase Activity of *Limosilactobacillus fermentum* U-21

**DOI:** 10.3390/biology13080592

**Published:** 2024-08-06

**Authors:** Rahaf N. Al Ebrahim, Maria G. Alekseeva, Sergey V. Bazhenov, Vadim V. Fomin, Dilara A. Mavletova, Andrey A. Nesterov, Elena U. Poluektova, Valeriy N. Danilenko, Ilya V. Manukhov

**Affiliations:** 1 Moscow Center for Advanced Studies, Kulakova Str. 20, 123592 Moscow, Russia; 2 Laboratory of Genetics of Microorganisms, Vavilov Institute of General Genetics Russian Academy of Sciences, 119991 Moscow, Russiaepolu@vigg.ru (E.U.P.);; 3 Laboratory of Microbiology, BIOTECH University, 125080 Moscow, Russia; 4 Institute of Environmental Engineering, RUDN University, 117198 Moscow, Russia; 5 Research Center of Neurology, 125367 Moscow, Russia

**Keywords:** *Limosilactobacillus fermentum*, chaperone, refolding, ClpL, ClpB, luciferase assay

## Abstract

**Simple Summary:**

The increase in the average life expectancy of people, the deterioration of the environment, and the stress of various etiologies, all these reasons contribute to the rising prevalence of Parkinson’s disease. With this, the relevance of developing drugs for the prevention and treatment of it increases. Parkinson’s disease is accompanied by a disruption of the protein structure and inflammatory processes. One of the approaches to combating this disease is the use of pharmabiotics based on bacteria that are able to produce enzymes that help maintain the native structure of proteins, i.e., possessing chaperone activity. Previously, *L. fermentum* U-21 has demonstrated the potential for use as a pharmabiotic. This study focuses on one of the proteins secreted by these bacteria, whose activity can help maintain the native state of proteins in patient tissues. We demonstrated the chaperone activity of the mixture of proteins secreted by *L. fermentum* U-21 cells and the presence of ClpL among these proteins. *In vivo* and *in vitro* tests showed the chaperone activity of ClpL. These findings indicate an involvement of the ClpL chaperone in the disaggregase activity and pharmabiotic properties of *L. fermentum* U-21.

**Abstract:**

The *L. fermentum* U-21 strain, known for secreting chaperones into the extracellular milieu, emerges as a promising candidate for the development of novel therapeutics termed disaggregases for Parkinson’s disease. Our study focuses on characterizing the secreted protein encoded by the C0965_000195 locus in the genome of this strain. Through sequence analysis and structural predictions, the protein encoded by C0965_000195 is identified as ClpL, homologs of which are known for their chaperone functions. The chaperone activity of ClpL from *L. fermentum* U-21 is investigated *in vivo* by assessing the refolding of luciferases with varying thermostabilities from *Aliivibrio fischeri* and *Photorhabdus luminescens* within *Escherichia coli* cells. The results indicate that the *clpL* gene from *L. fermentum* U-21 can compensate for the absence of the *clpB* gene, enhancing the refolding capacity of thermodenatured proteins in *clpB*-deficient cells. *In vitro* experiments demonstrate that both spent culture medium containing proteins secreted by *L. fermentum* U-21 cells, including ClpL, and purified heterologically expressed ClpL partially prevent the thermodenaturation of luciferases. The findings suggest that the ClpL protein from *L. fermentum* U-21, exhibiting disaggregase properties against aggregating proteins, may represent a key component contributing to the pharmabiotic attributes of this strain.

## 1. Introduction

ClpB protein, despite its name being derived from caseinolytic protease [1,2], is not a protease but rather an AAA ATPase chaperone. In *E. coli* cells, ClpB belongs to the HSP100 family of heat shock proteins, along with ClpA and ClpX [3]. While ClpA and ClpX are known chaperones [4,5], they can also form a complex with the protease subunit ClpP, determining substrate specificity in the proteolysis of cellular proteins by ClpAP and ClpXP complexes [6,7,8]. In contrast, ClpB does not interact with ClpP. ClpB is characterized by its interaction with the DnaKJ/GrpE chaperone complex [9]. Notably, a knockout of *clpB* results in a significant decrease in the DnaK-dependent refolding of bacterial luciferases [10]. Additionally, the Clp family is presented in Gram-positive bacteria by the ClpE, ClpC, and ClpL proteins, which differ in certain structural elements [11,12].

The bacterial proteins of the Clp family often play an important role in the interaction of bacterial cells with eukaryotic organisms. In particular, elevated plasma concentrations of bacterial ClpB protein have been observed in patients with eating disorders [13]. Studies have suggested that the bacterial protein ClpB acts as a conformational mimetic of α-MSH and may play a role in the transmission of satiety signals [14,15]. *L. fermentum* is a species commonly isolated from the gastrointestinal tract. Several *L. fermentum* strains possessing immunomodulatory, anti-inflammatory, antiviral, and anti-oxidative effects have shown potential as pharmabiotics in *in vitro* models, preclinical studies, and human trials [16,17].

The *L. fermentum* U-21 strain, isolated in the Laboratory of Microbial Genetics, Vavilov Institute of General Genetics RAS, has demonstrated high antioxidant activity in *in vitro* models using the oxidative stress inducer paraquat. *In vivo* experiments on animal models of Parkinson’s disease have shown that the administration of *L. fermentum* U-21 improves movement coordination, prevents the degradation of dopaminergic brain neurons, increases survival rates, preserves tyrosine hydroxylase-positive nerve fibers, and enhances the number of goblet cells in enteric nerve plexuses [18,19]. High biological activity was observed both in the 1-methyl-4-phenyl-1,2,3,6-tetrahydropyridine (MPTP)-induced and paraquat-induced Parkinsonism models. Taken together, these data demonstrate the unique antioxidant activity properties of this strain, allowing us to position it as a promising candidate for the development of pharmaceuticals aimed at preventing and treating various inflammatory conditions, such as Parkinsonism.

Previous research [20] has shown that the *L. fermentum* U-21 strain secretes a protein encoded by the *clp*-family gene in the locus C0965_000195, which is absent in *L. fermentum* strains 103 and 279, which lack antioxidant properties. This secreted protein, initially denoted as ClpB, was hypothesized to contribute to the pharmabiotic properties of U-21 by refolding proteins misfolded due to oxidative stress in various tissues of the animal body.

The present work aims to study the chaperone properties of this Clp protein in order to characterize it as a component of the disaggregase activity of *L. fermentum* U-21. The annotation of the gene located at locus C0965_000195 has been refined as *clpL*. The ability of *clpL* to accelerate the refolding of thermodenatured proteins was investigated *in vivo* using a luciferase model in *E. coli clpB^−^* cells, which exhibit a reduced refolding capacity. Additionally, *in vitro* experiments with purified *P. luminescens* luciferase explored the effect of the compounds and proteins from the spent *L. fermentum* U-21 culture medium (SCM) and separately purified ClpL on the thermodenaturation of luciferase.

## 2. Materials and Methods

### 2.1. Bacterial Strains and Plasmids

The following strains were used in the study:

- *L. fermentum* U-21 (collection number VKPM V-12075, NCBI Genome assembly ASM286982v2);
- *E. coli* SG20250 (Δ*lac*U169 *ara*D *flb*B *rel*A *clp*B^+^), and its insertion derivative SG22100 *clp*B::*kan*^−^ (kindly provided by S. Gottesman) [21];
- *E. coli* XL1-Blue (*rec*A1 *end*A1 *gyr*A96 *thi*-1 *hsd*R17 *sup*E44 *rel*A1 *lac* [F’*pro*AB *lac*IqZΔM15 Tn10 (Tet^r^)]) (Stratagene, La Jolla, CA, USA);
- *E. coli* BL21-Gold (DE3) (*F*^−^ *ompT dcm^+^* TetR *gal lon hsdSB(rB*^−^ *mB*^−^) *λ*(*DE3* [*lacI lacUV5-T7p07 ind1sam7 nin5*]) used for the biosynthesis of luciferase LuxAB and NADH-FMN oxidoreductases LuxG (obtained from VKPM);
- *E. coli* Nico21(DE3) can::CBD fhuA2 [lon] ompT gal (λ DE3) [dcm] arnA::CBD slyD::CBD glmS6Ala ∆hsdS λ DE3 = λ sBamHIo ∆EcoRI-B int::(lacI::PlacUV5::T7 gene1) i21 ∆nin5 (NEB strain catalog no. C2529H);
- *E. coli* TG1 (*thi rel*A *sup*E44 *hsd*R17 *hsd*M Δ(*lacpro*AB) [F’*tra*D36 *pro*AB *lac*IqZ ΔM15]) for plasmid preparation (obtained from VKPM).

A description of the plasmids used in this work is given in Table 1.

### 2.2. Cultivation Conditions

The *L. fermentum* U-21 strain was grown on the Man–Rogosa–Sharpe (MRS) medium (HiMedia) at 37 °C under partially anaerobic conditions (in a desiccator where oxygen was burned up by burning a candle). The *E. coli* strains were cultivated in Lysogeny Broth (LB) or on LB agar plates (1.5% (*w*/*v*) agar) [25]. The medium was supplemented with ampicillin (150 mg/L), tetracycline (10 mg/L), or chloramphenicol (10 mg/L) as needed. For blue–white screening during pUC19:clpL cloning, 50 µg/mL IPTG and X-gal were added into the medium.

### 2.3. DNA Manipulations

Genomic DNA of *L. fermentum* U-21 strain was obtained using the GenElute bacterial genomic DNA kit (Sigma Aldrich Inc., St. Louis, MO, USA). Plasmid DNA isolation, preparation of competent *E. coli* cells, and transformation were performed using standard methods [25]. The DNA fragment encoding ClpL*,* which was previously annotated as ATP-dependent Clp protease ATP-binding subunit ClpB [20], was amplified from the *L. fermentum* U-21 genomic DNA. The amplification was carried out using a Tersus Plus PCR kit (Evrogen, Moscow, Russia) with oligonucleotides ClpL-N and ClpL-C on a PTC-0150 minicycler (MJ Research Inc., Watertown, MA, USA). The amplified DNA fragment was then cloned into a pUC19 plasmid at the *Hind*III and *Xba*I restriction sites. To screen for recombinant clones containing the final plasmid pUC19:clpL, PCR was performed using M13dirShort and M13rev primers (Evrogen, Moscow, Russia).

For the preparation of the p15FisAB plasmid, based on p15 replicon, the *A. fischeri luxAB* genes were amplified from plasmid pF6. The resulting DNA fragment was then ligated with EcoRI-linearized p15Tc-lac vector using Gibson Assembly [26].

Similarly, p15XenAB plasmid based on p15 replicon was obtained for the expression of *P. luminescens luxAB* genes. The *lux* operon of *P. luminescens* from plasmid pXen7 was used as source of genes.

To construct the pABX-T7 plasmid, the *luxAB* fragment from pXen7 was amplified and cloned in BamHI-NcoI sites of the pET15b vector. This resulted in placing the *luxAB* genes downstream of the T7 promoter with the addition of the 6× His-tag at the N-terminus of LuxA.

pLuxG-T7 plasmid was prepared by linearizing the pET15b vector by NdeI restriction endonuclease, amplifying the *luxG* gene from the genomic DNA of *Vibrio aquamarinus* [27], and ligating the resulting fragments using Gibson Assembly.

For the creation of pET16b.clpL plasmid, the pET16b vector was linearized by NdeI and XhoI endonucleases. The *clpL* gene from pUC19:clpL was then amplified and ligated into the linearized pET16b vector.

The primers used in the study are presented in Table S1.

### 2.4. Expression of the ClpL Gene from L. fermentum U-21 Strain in E. coli

Transformant clones of *E. coli* XL1-Blue strain containing recombinant plasmid pUC19:clpL were seeded in liquid LB medium supplemented with ampicillin and grown in a shaker-incubator for 18 h at 37 °C, 250 rpm. Then, the overnight culture was diluted 1:100 with fresh LB and grown in a shaker-incubator at 37 °C, 250 rpm. Probes were collected at specific time points: 2, 3, 4, 5, and 24 h of incubation. The culture’s optical density (OD) was measured at a wavelength of 600 nm using a spectrophotometer.

To investigate *clpL* gene expression, cells were precipitated by centrifugation and suspended in a sample buffer containing 62.5 mM Tris-HCl (pH 6.8), 5% glycerol, 2% 2-mercaptoethanol, 0.1% SDS, and 0.001% bromphenol blue. The suspended cells were heated at 95 °C for 10 min. The proteins were then analyzed by SDS-PAGE. *E. coli* XL1-Blue cells containing plasmid pUC19 without insertion were used as a negative control.

### 2.5. In Vivo Luminescence Measurement

Luminescent cells at the early stationary growth phase were suspended in LB medium to achieve a final OD of ~0.01. *E. coli* cells containing the corresponding genes were incubated in a water bath at 44 °C for 9–15 min for *in vivo* thermal inactivation of *A. fischeri* luciferase, or at 47 °C for 20 min for inactivation of *P. luminescens* luciferase. To halt the production of luciferase and heat shock proteins, chloramphenicol was added to the cell suspension to a final concentration of 167 μg/mL. The cells were then transferred to a lower temperature of 22 °C to allow for subsequent refolding. Activation of luciferase reaction was carried out by adding 0.001% decanal. Luminescence measurements in Relative Light Units (RLUs) were performed using a Biotox-7BM luminometer (BioPhysTech, Dolgoprudny, Russia).

### 2.6. Biosynthesis, Isolation, and Purification of P. luminescens Luciferase, V. aquamarinus LuxG, and L. fermentum U-21 ClpL

The expression of *P. luminescens* luciferase LuxAB in *E. coli* BL21-Gold (DE3) and *L. fermentum* U-21 ClpL in *E. coli* Nico21(DE3) with subsequent purification was carried out using the following method. Cells were cultured in baffled shake flasks containing LB medium supplemented with 150 mg/L ampicillin. Protein expression was induced by adding 1 mM IPTG (Isopropyl β-D-1-thiogalactopyranoside) and continued for 22 h at 20 °C for LuxAB or 1.5 h at 26 °C for ClpL. Harvested cells were resuspended in phosphate-buffered saline with the addition of 1 mM phenylmethylsulfonyl fluoride (PMSF) as a protease inhibitor. Cell disruption was performed using an M-110P Lab Homogenizer (Microfluidics, Newton, MA, USA) at a pressure of 25,000 psi. The lysate was clarified by removing the cell membrane fraction through ultracentrifugation at 40,000× *g* for 1 h at 10 °C. The supernatant obtained after ultracentrifugation was incubated with Ni-nitrilotriacetic acid (Ni-NTA) resin (Qiagen, Hilden, Germany) on a rocker for 1 h at 4 °C. The Ni-NTA resin and supernatant were loaded onto a gravity flow column and washed with buffer containing 150 mM NaCl and 50 mM Tris-HCl (pH 8.0), supplemented with 20 mM imidazole. The proteins of interest were then eluted from the column using a buffer containing 50–500 mM imidazole, 150 mM NaCl, and 50 mM Tris-HCl (pH 8.0). To remove imidazole from the eluted fractions, centrifugation at 3300× *g* was performed using 10 kDa pore concentrators. The eluted proteins were then transferred to fresh buffer without imidazole. The purity of the obtained ClpL was assessed by SDS-PAGE as shown in Figure S3, and it was about 90% according to ImageJ IJ 1.46r analysis.

For the purification of LuxG, a similar method was employed with some modifications:

LuxG protein was predominantly in an aggregated state, and, hence, after the cell lysis, it was dissolved in 2M urea. The dissolved protein was then centrifuged at 15,000× *g*, and the supernatant obtained was applied to the Ni-NTA resin. The column was washed as described previously. The resulting LuxG protein, with a concentration of approximately 0.2 mg/mL and exhibiting enzymatic activity, was further used for the restoration of FMN (Flavin Mononucleotide) in the luciferase reaction.

### 2.7. Spent L. fermentum U-21 Culture Medium

To prepare a spent culture medium (SCM), *L. fermentum* U-21 cells underwent a 24 h cultivation process, followed by centrifugation at 7000 rpm and filtration through a 0.22 µm filter. To prepare the SCM with inactivated proteins, the SCM obtained at the previous step was heated to 100 °C for 30 min. The method for mass-spectrometry analysis of SCM is described in Supplementary Text S1.

### 2.8. Measurement of P. luminescens Luciferase Activity In Vitro

The assessment of *P. luminescens* luciferase activity *in vitro* involved the addition of purified LuxAB protein to a reaction solution composed of 50 mM Tris-HCl (pH 8.0) and 150 mM NaCl buffer at a final concentration of 40 μg/mL. The reaction mixture contained 20 μM FMN, 0.2 mM NADH, 20 μg/mL LuxG, and 1 mM ATP. This solution was then divided into multiple samples for testing. In the first sample, 10 μL of SCM containing ClpL was added. The second sample incorporated 10 μL of SCM containing ClpL that had been inactivated at 100 °C. The third sample, which served as a control experiment, was without the addition of SCM. Varying concentrations of purified ClpL protein were added to the remaining samples. Each sample was heated at 45 °C for 9–11 min. Following thermoinactivation, refolding was monitored through periodic sampling with luminescence measurements conducted at room temperature. The luminescence reaction was triggered by the addition of the luciferase substrate decanal at a final concentration of 0.001% (*v*/*v*). Luminescence measurements were performed using Biotox-72K luminometer (Kalinichenko, Moscow, Russia).

### 2.9. Phylogenetic and Molecular Evolutionary Analysis

Phylogenetic and molecular evolutionary analyses were carried out using MEGA11 with the MUSCLE algorithm [28]. Phylogenetic tree was built using the neighbor-joining method incorporated in MEGA11. For the phylogeny analysis, reference sequences of Clp proteins from various organisms were retrieved from GenBank, with their respective GenBank IDs within the text (https://www.ncbi.nlm.nih.gov/genbank/, accessed on 7 May 2024).

## 3. Results

### 3.1. Phylogenetic Analysis of C0965_000195 L. fermentum U-21

**Figure 1:**
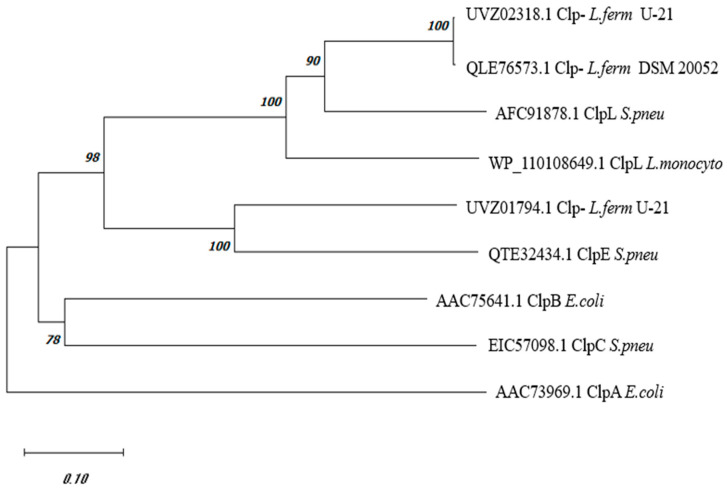
Phylogenetic tree of Clp proteins from *L. fermentum* and reference sequences of Clp proteins with a confirmed function from other organisms. The sequences of Clp proteins: *L. fermentum* U-21 (UVZ02318.1 and UVZ01794.1), *L. fermentum* DSM 20052 (QLE76573.1), *E. coli* MG1655 ClpA (AAC73969.1) and ClpB (AAC75641.1), *S. pneumoniae* ClpC (EIC57098.1), ClpE (QTE32434.1) and ClpL (AFC91878.1), and *L. monocytogenes* ClpL (WP_110108649.1).

The comparative analysis of the amino acid sequence of the *L. fermentum* U-21 UVZ02318.1 protein, derived from the gene expression at the C0965_000195 locus, annotated as “ATP-dependent Clp protease ATP-binding subunit or ClpB” [20], revealed almost equal similarities with ClpA and ClpB proteins from *E. coli* MG1655, exhibiting approximately 39.51% and 41.96% identical amino acids, respectively. Moreover, intra-species comparison between ClpA and ClpB from *E. coli* demonstrated around 40.1% identical amino acids. Notably, other Clp-family proteins such as ClpC, ClpE, and ClpL were predominantly found in Gram-positive bacteria. A phylogenetic tree constructed using the amino acid sequences of UVZ02318.1 *L. fermentum* U-21, along with a series of Clp proteins from *Streptococcus pneumonia* and *Listeria monocytogenes* whose functions are described in the literature [8,11,12,29,30], is shown in Figure 1. Notably, the currently unannotated sequence QLE76573.1 from the type strain *L. fermentum* DSM 20052, sharing 99.57% identical amino acids with UVZ02318.1, was added in the tree for reference. Additionally, the sequence UVZ01794.1 of *L. fermentum* U-21 annotated as ClpA, sharing 50% identical amino acids with the UVZ02318.1, was also depicted in the tree.

As can be seen from the data in Figure 1, the phylogenetic tree allows one to assign with some certainty UVZ02318.1 and QLE76573.1 proteins from *L. fermentum*, as ClpL, and UVZ01794.1 protein, as ClpE.

**Figure 2:**
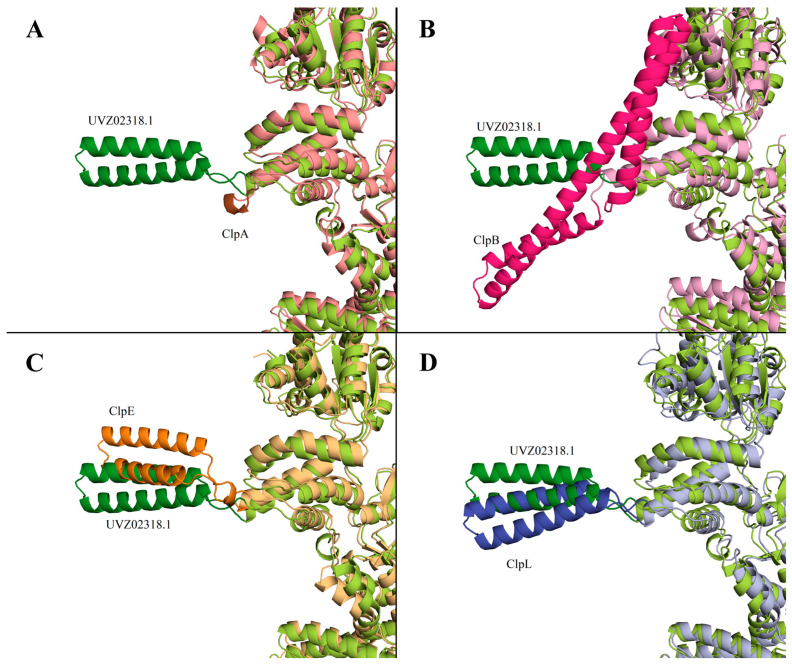
AlphaFold model of UVZ02318.1 (“limon” color, M-domain highlighted in darker “forest” color) in pairwise comparison with AlphaFold structures of the following proteins: ClpA *E. coli* (panel (**A**); color “salmon”, M-domain—“brown”), ClpB *E. coli* (panel (**B**); “pink”, “hotpink”), ClpE *S. pneumonia* (panel (**C**); “lightorange”, “tv_orange”), and ClpL *S. pneumonia* (panel (**D**); “lightblue”, “tv_blue”).

It was described in [31] that a distinctive feature of ClpB protein is the presence of M-domain in the structure (396-512 aa for WP_011228712). No homologous sequence corresponding to the M-domain of ClpB was found in UVZ02318.1. It should be noted that in ClpA, the M-domain is completely absent, while in ClpC, ClpE, and ClpL proteins, this domain is present but has a smaller size, and its structure differs significantly from the ClpB M-domain. Figure 2 shows a comparison of the AlphaFold models of ClpA, ClpB, ClpL, and ClpE proteins with the AlphaFold-predicted structure of Clp-protein UVZ02318.1. The M-domain is highlighted in more intense colors.

The comparison of the structures of the Clp-family proteins shows a characteristically significant difference in the M-domains with a general similarity of the main part of the protein. Based on the alignment with other Clp-family proteins, UVZ02318.1 is most closely related to ClpL, with some resemblance to ClpE.

In all, the bioinformatic analysis of the UVZ02318.1 protein encoded by the gene at locus C0965_000195 demonstrates its classification within the Clp-protein family as ClpL. Hence, the UVZ02318.1 protein is denoted as *L. fermentum* U-21 ClpL in our investigation.

### 3.2. Gene Cloning and Expression and Chaperone Activity Investigation of the L. fermentum U-21 ClpL Protein in E. coli Cells

To investigate the chaperone activity of ClpL from *L. fermentum* U-21, the *clpL* gene was cloned and expressed in a heterologous system of *E. coli* cells. To analyze the expression of the *clpL* gene in *E. coli*, the XL1-Blue strain containing recombinant plasmid pUC19:clpL was grown in liquid LB medium. The soluble fraction of proteins was analyzed by SDS-PAGE (Figure S1). During the stationary growth phase and at the beginning of the logarithmic growth phase (after 2 h), an additional protein fraction with a molecular mass of about 77.5 kDa was observed in *E. coli* cells containing the pUC19:clpL plasmid. This molecular mass corresponds to the calculated molecular mass of the ClpL protein in combination with the molecular mass of the linker protein of the pUC19 plasmid. Mass-spectrometric analysis confirmed that this protein is an “ATP-dependent Clp protease ATP-binding subunit” ClpL of *L. fermentum* U-21. Furthermore, the presence of a high content of ClpL protein led to a delay in the transition to the exponential growth phase of strain XL-1 pUC19:clpL compared to the control strain XL-1 pUC19 (Figure S2).

**Figure 3:**
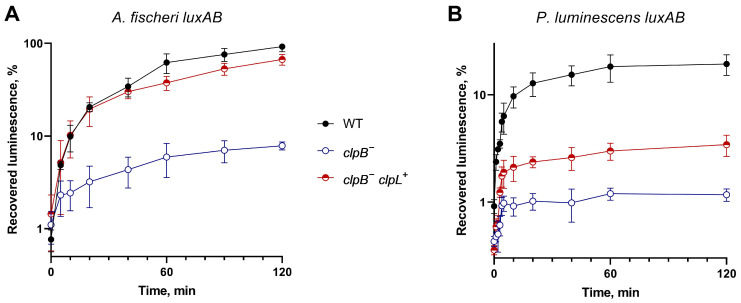
Effect of the *clp*B::*kan* mutation and *L. fermentum* U-21 *clpL* gene introduction on the kinetics and level of refolding of heat-inactivated bacterial luciferases from *A. fischeri* (panel (**A**)) and *P. luminescens* (panel (**B**)) in *E. coli* cells. Inactivation of LuxAB *A. fischeri* was conducted by heating to 44 °C for 10 min, and LuxAB *P. luminescens* were inactivated by heating to 47 °C for 20 min. Ordinate—recovered luciferase activity relative to the initial activity before inactivation; abscissa—time of refolding at 22 °C after inactivation. Means and error bars were calculated from three independent replicates. At 120 min, measurements for “WT”, “*clpB*^−^”, and “*clpB*^−^ *clpL*^+^” reliably differ with *p*-value < 0.01 for all, except “WT” and “*clpB*^−^ *clpL*^+^” *A. fischeri*, which differ with *p*-value ~0.02.

*E. coli* cells mutant in *clpA* and *clpB* genes have a low ability to refold heat-inactivated proteins [10]. Hence, these *E. coli* strains are ideal model hosts for the investigation of the ability of various heterologous proteins to compensate for the chaperone function. To assess the ability of ClpL to compensate the ClpB absence in refolding denatured proteins, *E. coli* SG20250 and its insertion derivative SG22100 *clpB::kan^−^* were used as hosts. The recovery of luminescence after the heat inactivation of the luciferases was measured in cells harboring p15XenAB or p15FisAB plasmids separately or in combination with pUC19:clpL (Figure 3). pUC19:clpL drives the heterologous expression of *clpL* from *L. fermentum* U-21; p15XenAB and p15FisAB are compatible with pUC19:*clpL* and drive the heterologous expression of *luxAB* from *P. luminescens* and *A. fischeri*, correspondently.

The data presented in Figure 3 demonstrate that pUC19:clpL partially compensates for the deletion of *clpB* during the refolding of luciferases. The compensation is more pronounced for the thermolabile luciferase of *A. fischeri* and less prominent for the more thermostable luciferase of *P. luminescens.* LuxAB *P. luminescens* has poor ability to refold in *E. coli* in general. These findings are consistent with previous data [10] that indicate the importance of ClpB in DnaK-dependent refolding, with *clpB* deletion resulting in a 10-fold reduction in refolding efficiency. The ability of *clpL* from U-21 to compensate for the absence of *clpB* suggests a potential interaction between *L. fermentum* U-21 ClpL and the DnaKJE chaperone complex of *E. coli*.

### 3.3. Investigation of the Chaperone Activity of L. fermentum U-21 SCM In Vitro

**Figure 4:**
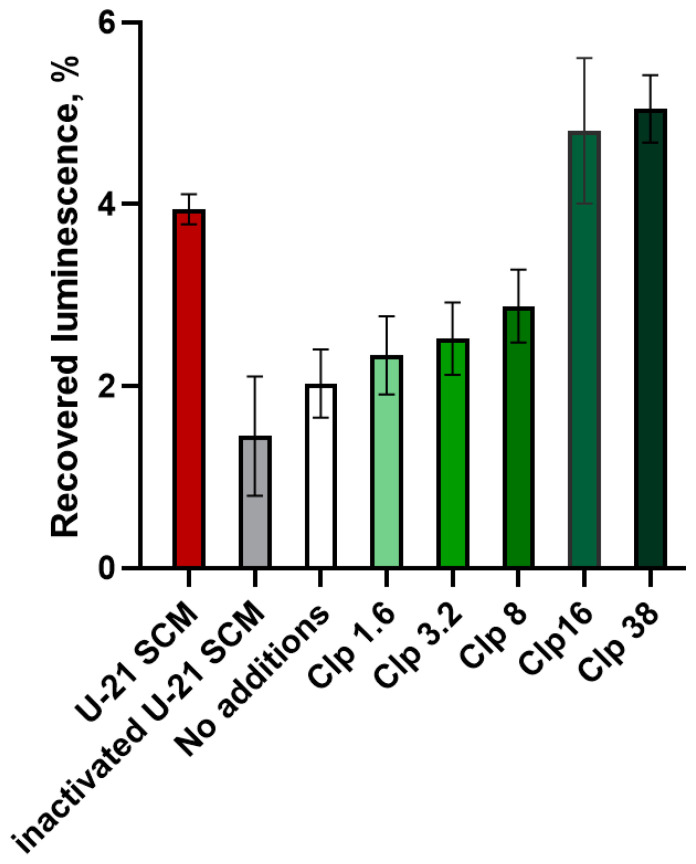
Recovered activity of thermoinactivated *P. luminescens* luciferase after refolding *in vitro* dependent on the presence of the SCM of *L. fermentum* U-21 or purified ClpL. SCM was added, representing 10% of the final reaction volume, with a ClpL concentration of 1.6–38 µg/mL.

The SCM of *L. fermentum* U-21 cells, known to contain ClpL protein [20], was analyzed for its chaperone activity. Electrophoresis coupled with mass-spectrometry analysis revealed the presence of various proteins, including chaperones ClpL, DnaK, DnaJ, GroEL, GroES, ClpX, and other Clp-family members (Table S2). The effect of the SCM on the activity of the thermally denatured luciferase was tested *in vitro* using purified *P. luminescens* LuxAB. NADH, FMN, and LuxG were added to the reaction medium to maintain the pool of reduced FMNH_2_, which is a co-factor for the luciferase. For the identification of the role of proteins from the SCM in the refolding of luciferase, three types of samples were compared: luciferase reaction mixture without SCM, with the addition of SCM (final concentration of 10%), and with the addition of boiled SCM in the same amount. As a positive control, a purified ClpL protein from *L. fermentum* U-21, expressed in *E. coli* cells, was used. After the thermal inactivation of luciferase (45 °C for 9–11 min) and 4 min of refolding at room temperature, the luciferase reaction was triggered by the addition of decanal, and the luminescence was measured. The experiment was conducted in three independent biological replicates. Figure 4 shows the typical results with error bars corresponding to the measurement error (standard deviation) within a single replicate. From one experiment to another, the overall refolding efficacy varies, but each time the SCM and high concentrations of the purified ClpL enhance the luminescence approximately twice, while the boiled SCM does not have a significant effect.

Figure 4 presents the efficacy of luminescence recovery after thermal inactivation and the subsequent refolding of luciferase. The control sample without the addition of SCM showed the recovery of only about 2% of the initial luminescence. The addition of SCM to the sample increased the luminescence level by an average of two times. Boiled SCM had a slight negative effect on luminescence, but this effect was not statistically significant. Furthermore, the addition of purified ClpL protein prevented luciferase thermal inactivation in a dose-dependent manner. The effect became statistically significant when the concentration of ClpL was 38 µg/mL (paired *t*-test based on independent biological replicates, *p*-value < 0.01).

## 4. Discussion

Bioinformatics analysis comparing the amino acid sequence encoded by the gene located at locus “C0965_000195” in the *L. fermentum* U-21 genome with known Clp-family proteins (ClpA, ClpB, ClpC, ClpE, and ClpL) revealed a high similarity to ClpL (Figure 1 and Figure 2). This, therefore, allows us to assign the locus gene to *clpL*, which was first described for plasmids containing a transposon-like structure [32]. The presence of a specific M-domain characteristic of the ClpL protein was observed in the predicted protein structure (Figure 2), and the confidence scores obtained from AlphaFold models indicated the reliability of the predicted structures. Presented in Figure 2, AlphaFold-produced fragments have a per-residue confidence score (pLDDT) between 70 and 100 with the link of M-domain to the rest of the protein with pLDDT between 50 and 70. The intermediate confidence scores indicate the flexibility of that link, which could be the reason for the missing M-domains in most of the X-ray structures of Clp proteins. ClpL is known to exhibit chaperone activity [33], lacks the motif for binding to the protease subunit of ClpP [34], and does not require DnaKJ-type auxiliaries for its chaperone function in *in vitro* experiments [29].

In this study, we successfully cloned, expressed, and functionally characterized the *clpL* gene from *L. fermentum* U-21 in *E. coli* cells. High levels of ClpL were observed during the late exponential and stationary growth phases. The disappearance of the target band on SDS-PAGE between 3 and 5 h of growth (Figure S1) likely resulted from the proteolytic degradation of ClpL within *E. coli* cells. This hypothesis was supported by the observation of smaller fragments in the fraction of purified ClpL from *E. coli* NiCo21(DE3) pET16b.clpL cells after prolonged expression. When expression lasted 6 h, a significant degradation of ClpL into smaller fragments was observed (Figure S3, lane 6), whereas 1.5 h of induction allows the isolation of the full-length protein. Moreover, cells expressing ClpL exhibited a significant delay in transition to the exponential growth phase. We demonstrate the *in vivo* chaperone activity of ClpL by its ability to compensate for the absence of the *clpB* gene in *E. coli* cells. At the same time, we found no compensation for refolding in *clpA*^−^ mutant cells (data not presented), suggesting that ClpL may operate through mechanisms similar to ClpB rather than ClpA.

Our *in vitro* assays further confirmed the chaperone activity of ClpL. The SCM of *L. fermentum* U-21, which contains various proteins including ClpL, significantly enhanced *P. luminescens* luciferase activity after thermal inactivation. The addition of the purified ClpL protein alone also prevented luciferase thermal inactivation in a dose-dependent manner. These findings are consistent with recent research [30] on the chaperone activity of ClpL from *L. monocytogenes*. The ability of ClpL to prevent protein aggregation and maintain protein activity suggests its potential application in addressing protein misfolding- and aggregation-related disorders, such as amyloid diseases including [30] Alzheimer’s, Parkinson’s, and Huntington’s diseases. Disaggregases are being intensively investigated as potential drugs targeting protein misfolding and aggregation [35,36]. Agents able to disaggregate preformed amyloids have been classified as molecular chaperones (e.g., Hsp70 and Hsp90), chemical chaperones (bile acids, steroid hormones, and trehalose), and pharmacological chaperones (amino acid derivatives, benzophenone, and tetracycline) [37]. The bacterial ATPases of the Clp proteins family have been suggested to be used for such purposes [38].

The compensation of *clpB* deficit in *E. coli* by *clpL* from *L. fermentum* suggests that ClpL may interact with heterologous environments, including other chaperone systems such as DnaKJ or even eukaryotic chaperones. This potential interaction may be important for the pharmacobiological effects of the proteins secreted by U-21 cells. *L. fermentum* U-21 was shown to act as pharmabiotic [39], which can reduce pro-inflammatory changes in rat models of Parkinson’s disease [40]. Certain bacterial strains, including *Lactobacillus, Bifidobacterium,* and *Faecalibacterium*, have shown the ability to alleviate symptoms of neurodegenerative diseases in animal models [41,42]. Clinical trials also support the potential of pharmabiotic in ameliorating manifestations of nigral dopaminergic neuronal death and motor deficits and regulating dopamine pathways in individuals with Parkinson’s disease [43,44]. However, the specific components of bacterial cells responsible for these effects and the underlying mechanisms remain unclear [39,40].

## 5. Conclusions

Our study provides the functional characteristics of the ClpL protein from *L. fermentum* U-21. The cloning, expression, and *in vivo* and *in vitro* assays demonstrated its chaperone activity. Our results suggest that the anti-Parkinsonian properties of the *L. fermentum* U-21 strain are determined, in part, by the ClpL protein and its ability to refold protein aggregates.

## Figures and Tables

**Table 1:** Description of plasmids.

Name	Description	Source
p15Tc-lac	Expression vector. P*tac*-MCS, *lacI*, ori-p15A, and Tc^r^	[22]
pF6	pACYC184 vector with *luxABE* from *A. fischeri* under P*lac* promoter, Cm^r^	[23]
p15FisAB	p15Tc-lac with *luxAB* from *A. fischeri* under P*tac* promoter, Tc^r^	This study
pXen7	pUC18 with *luxCDABE* operon from *P. luminescens* under control of its own promoter, Ap^r^	[24]
p15XenAB	p15Tc-lac with *luxAB* from *P. luminescens* under P*tac* promoter, Tc^r^	This study
pUC19:clpL	pUC19 vector (Sigma-Aldrich Inc., USA) with *clpL* from *L. fermentum* U-21 under P*lac* promoter, Ap^r^	This study
pABX-T7	pET15b with *luxAB* from *P. luminescens* under T7 promoter, Ap^r^	This study
pLuxG-T7	pET15b with *luxG* from *V. aquamarinus* under T7 promoter, Ap^r^	This study
pET16b.clpL	pET16b vector (Novagen, Madison, WI, USA) with *clpL* from *L. fermentum* U-21 under T7 promoter, Ap^r^	This study

## Data Availability

Available data are presented in the manuscript and in the Supplementary Materials.

## Supplementary Materials

The following supporting information can be downloaded at: https://www.mdpi.com/article/10.3390/biology13080592/s1. Table S1: Primers used in the study; Text S1: Fingerprint analysis of the proteins; Table S2: Chaperone proteins in the spent culture medium (SCM) after cultivation of *L. fermentum* U-21; Figure S1: Electrophoretogram of the soluble protein fraction of *E. coli* XL1-Blue strains containing pUC19 (lanes 1, 3, 5, 7, 9, and 11) and pUC19:clpL (lanes 2, 4, 6, 8, 10, and 12) plasmids after 2, 3, 4, 5, 18 and 24 h of growth; Figure S2: Growth curves of *E. coli* XL1-Blue strains containing pUC19 and pUC19:clpL plasmids; Figure S3: Electrophoretogram of the lysated Nico21(DE3) cells containing pET16b.clpL before induction (lane 1) and after induction for 45 and 90 min (lanes 2 and 3). The next lanes present ClpL protein purified by affinity chromatography after 1.5 h of expression (lanes 4 and 5) and after overnight expression (lane 6). Ref. [45] is cited within the Supporting Information.
