# Supplementary material for: ClpL Chaperone as a Possible Component of the Disaggregase Activity of Limosilactobacillus fermentum U-21
Source: Biology (Basel). 2024 Aug 6;13(8):592. doi: 10.3390/biology13080592 (PMC11351774; doi:10.3390/biology13080592)
Supplement: Supplementary file 1 [file biology-13-00592-s001.zip › biology-3110215-supplementary.pdf]

Supplementary Material

Table S1. Primers used in the study.

**Name**	**Sequence**	**Note**
ClpL-N	CGCCAAGCTTGATGGCACGAATTCCAGTAGATC	For pUC19:clpL construction
ClpL-C	ATCCTCTAGAGTTTACTTTGCTTGTTCAATCACGAC	
M13dirShort	GTAAAACGACGGCCAGT	For plasmid pUC19:clpL sequencing
M13rev	AGCGGATAACAATTTCACACAGGA	
luxAD	GCCATGGGCCATCATCATCATCATCACAGCGGCAGCGGCATGAAATTTGGAAACTTTTTGCTTAC	For pABX-T7 construction
luxBR	GTTGGATCCATATTCTTTTACTACATGTGGTACT	
luxGD	CCGCGCGGCAGCCATATGTTATGTACGGTAGAAAAAAATAGAACC	For pLuxG-T7 construction
luxGR	AGCCGGATCCTCGAGCAGTTATAGGTAAGCGAATGCGTCAGC	
FshA.p15.D	atttcacacaggaaacagaattAAAGGAATAGAGTATGAAGTTTGGA	For p15FisAB construction
FshB.p15.R	cctagtataggggacatgTTATGGTAAATTCATTTCGATTTTTTG	
XenAD-p15Tc	GGATAACAATTTCACACAGGAAACAGAATTCATGAAATTTGGAAACTTTTTGCTT	For p15XenAB construction
XenBR-p15Tc	CGGGTACCTAGTATAGGGGACATGAATTCCTTTTACTACATGTGGTACTTTTTAATA	
ClpL-pET-N	TCGTCATATGATGGCACGAATTCCAGTAGATC	pET16b.clpL
ClpL-pET-C	GATCCTCGAGTTACTTTGCTTGTTCAATCACGAC	

* The sites for restriction endonucleases are underlined.

Text S1. Fingerprint analysis of the proteins

The HPLC-MS/MS was performed at the ‘Human Proteome’ core facility center of the Institute of Biomedical Chemistry (Moscow, Russia).

One microgram of peptides in a volume of 1-4 µl was loaded onto the Acclaim µ-Precolumn (0.5 mm x 3 mm, 5 µm particle size, Thermo Scientific) at a flow rate of 10 µL/min for 4 min in an isocratic

mode of Mobile Phase C (2% acetonitrile, 0.1% formic acid). Then the peptides were separated with high-performance liquid chromatography (HPLC, Ultimate 3000 Nano LC System, Thermo Scientific, Rockwell, IL, USA) in a 20-cm long C18 column (Peaky, inner diameter of 100 µm, Molecta, Russia). The peptides were eluted with a gradient of buffer B (80% acetonitrile, 0.1% formic acid) at a flow rate of 0.3 µL/min. MS analysis was performed at least in triplicate with a Q Exactive HF mass spectrometer (Q Exactive HF Hybrid Quadrupole-OrbitrapTM Mass spectrometer, Thermo Fisher Scientific, Rockwell, IL, USA).

Raw MS data files were analyzed using the MaxQuant search engine (v.2.0.3.0) with the build-in Andromeda algorithm [1]. The UniProt FASTA database for *Limosilactobacillus fermentum* (June, 2022) concatenated with a reverse decoy database was used for proteins identification. Trypsin was specified as cleavage enzyme allowing up to two missing cleavages.

Table S2. Chaperone proteins in the spent culture medium (SCM) after cultivation of *L. fermentum* U-21

UniProt Accession	Locus Tag	Protein name	Mol. weight [kDa]
A0A2M8MTZ1	C0965_RS02075	Co-chaperonin GroES	9.9101
A0A2K2THG6	C0965_RS07720	Hsp20/alpha crystallin family protein	16.668
A0A2M8MTX9	C0965_RS02080	Chaperonin GroEL	56.856
A0A0F4HC42	C0965_RS02280	ATP-dependent Clp protease proteolytic subunit	21.48
A0A1L7GSN9	C0965_RS04410	Chaperone protein DnaK	67.123
**A0A855ZMN6**	C0965_RS00195	**ATP-dependent Clp protease ATP-binding subunit**	**76.678**
A0A2V2D4Q2	C0965_RS07895	ATP-dependent Clp protease ATP-binding subunit	82.93
A0A8F1XU77	C0965_RS03700	ATP-dependent Clp protease ATP-binding subunit ClpX	45.758
A0A843REA3	C0965_RS01400	33 kDa chaperonin	31.587
A0A0F4HBZ3	C0965_RS01395	ATP-dependent zinc metalloprotease FtsH	79.419
A0A1L7GSR0	C0965_RS04415	Chaperone protein DnaJ	41.421
A0A6D1XSK2	C0965_RS08845	ATP-dependent Clp protease ATP-binding subunit	92.9

* The ClpL protein discussed in the article is highlighted in bold.

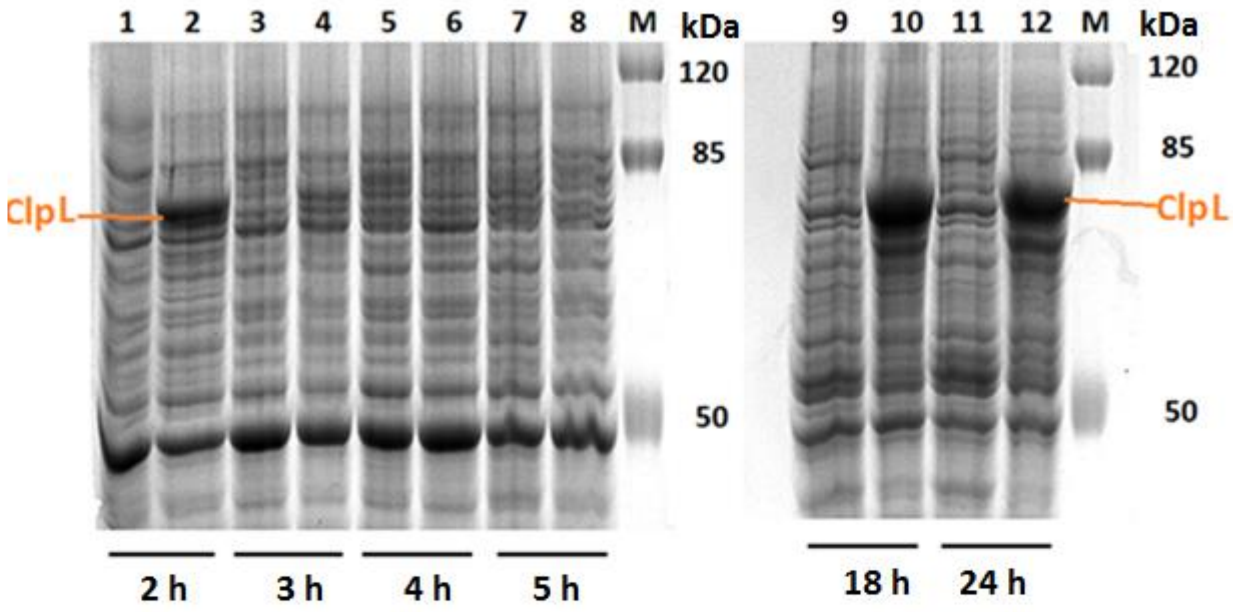

Figure S1. Electrophoretogram of the soluble protein fraction of *E. coli* XL1-Blue strains containing pUC19 (lanes 1, 3, 5, 7, 9,11) and pUC19:clpL (lanes 2, 4, 6, 8, 10, 12) plasmids after 2, 3, 4, 5, 18 and 24 hours of growth.

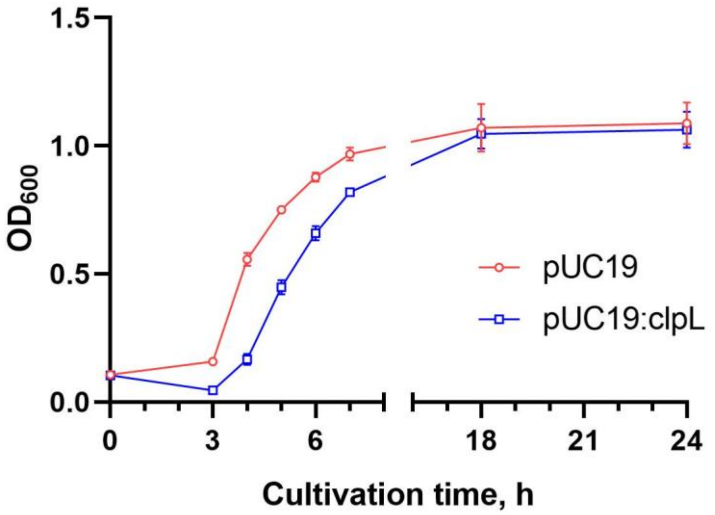

Figure S2. Growth curves of *E. coli* XL1-Blue strains containing pUC19 and pUC19:*clpL* plasmids.

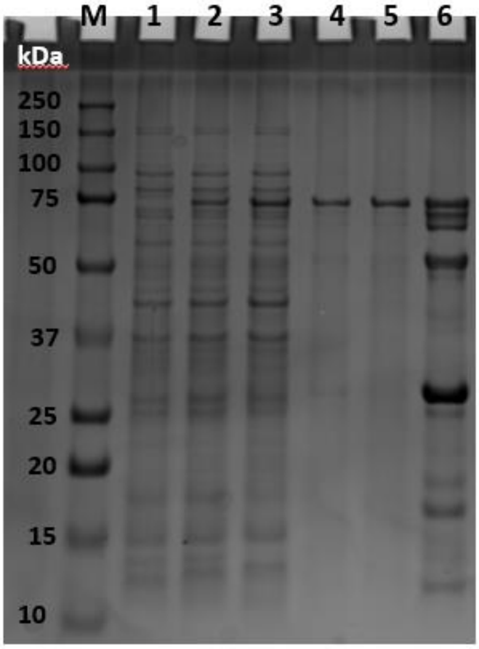

Figure S3. Electrophoretogram of the lysated Nico21(DE3) cells containing pET16b.clpL before induction (lane 1), after induction for 45 and 90 minutes (lanes 2 and 3). Next lanes present ClpL protein purified by affinity chromatography after 1.5 hours of expression(lanes 4,5) and after overnight expression (lane 6). According to ImageJ analysis, the protein purity in lanes 4 and 5 is approximately 93% and 97%, respectively.

REFERENCES

1. Tyanova, S.; Temu, T.; Cox, J. The MaxQuant Computational Platform for Mass Spectrometry-Based Shotgun Proteomics. *Nat Protoc* **2016**, *11*, 2301–2319, doi:10.1038/nprot.2016.136.
